# Getting It Right: Being Smarter about Clinical Trials

**DOI:** 10.1371/journal.pmed.0030144

**Published:** 2006-04-18

**Authors:** Barnett S Kramer, Joan Wilentz, Duane Alexander, John Burklow, Lawrence M Friedman, Richard Hodes, Ruth Kirschstein, Amy Patterson, Griffin Rodgers, Stephen E Straus

## Abstract

A major NIH meeting led to recommendations for conducting better clinical trials.

Concerns about adverse events, including deaths, in recent large clinical trials, both publicly and privately sponsored, prompted Elias A. Zerhouni, Director, National Institutes of Health (NIH) to convene a meeting at the NIH on January 11–12, 2005, to discuss “Moving from Observational Studies to Clinical Trials: Why Do We Sometimes Get It Wrong?” (a detailed summary and video archive of the meeting are available at http://www.meetinglink.org/omar/ct/index.htm). “It is time for an ‘M and M’ [Morbidity and Mortality] conference [on medical evidence],” Zerhouni said at the meeting. He challenged attendees to develop innovative ideas to aid decision and policy making, commenting that the credibility of the scientific enterprise was at stake. “Forty percent of science news relates to health or medicine,” he noted, “and we are seeing a gradual erosion of public trust.”

Experts in trial design and analysis reviewed sources of error that can affect clinical investigations from early observational studies to Phase 3 clinical trials. Speakers provided telling examples of biased observational data leading to unnecessary clinical trials, poor trial design and analysis, and misleading communication of results. Participants also proposed ways to maximize the quality of evidence available and considered the impact of the “-omics” revolution on the design of future clinical trials. Ample time was allowed for discussion, allowing the authors, who constituted the Planning Committee, to generate the recommendations (listed in Box 1) that serve as the organizational framework for this article.

## Recommendation 1: Be Aware of and Eliminate Bias and Confounders, to the Extent Possible

Among the most recent and notable clinical trials that called prior observational evidence into question was the NIH Women's Health Initiative clinical trial of combined estrogen and progestin hormone replacement therapy (HRT). In July 2002, the NIH halted the trial because statistical analysis indicated evidence of increased risk of breast cancer, heart disease, and stroke among postmenopausal women taking the combined estrogen–progestin regimen [ 1, 2]. In this case, conventional wisdom, based primarily on observational studies, so strongly supported the view that HRT would lower (rather than increase) the risk of heart disease, stroke, and dementia in postmenopausal women, as well as prevent hip fracture, that critics protested that NIH was wasting money on a Phase 3 clinical trial—or worse, that the trial was unethical because half the women would not receive the “known benefits” of HRT. But many at the meeting (for one example, see [ 3]) commented that there was selection bias in the observational studies. The women who were taking HRT in those studies were fundamentally different from those enrolled in this trial; they were generally leaner, less likely to smoke, better educated, more likely to exercise, and more likely to seek medical care than those who did not take HRT. Moreover, when the United States Preventive Services Task Force (an independent panel funded by the Agency for Healthcare Research and Quality) conducted a systematic review of HRT studies, it found “insufficient evidence” to support long-term use of HRT for chronic disease prevention [ 4]. (Following the results of the Women's Health Initiative trial, the panel recommended against use of combined HRT for the prevention of chronic disease.)

Many of these same issues of bias and confounders arose in “M and M” reviews of other clinical evidence. Based on reports that people eating foods rich in beta-carotene had lower risks of cancer, and the assumption that it was the antioxidant effects of the beta-carotene or of carotenoids in general that were responsible, at least four randomized clinical trials were conducted in the US and abroad [ 5–8]. Overall, the trials showed no benefit of beta-carotene in well-nourished populations, while some studies actually showed increased risk of lung cancer incidence and mortality in smokers exposed to the nutrient.

The beta-carotene trials overturned powerful intuitions about the role of specific nutrients in cancer prevention. Workshop participants also voiced concern about unwarranted assumptions that could lead to delays in conducting a trial. This was the case for beta-blockers, which for over a decade were not prescribed in cases of chronic heart failure because it was assumed that the drug's actions in lowering blood pressure and slowing heart rate would worsen heart failure. In fact, the drugs are beneficial. They have now been proven in randomized controlled trials to decrease mortality in patients who suffer chronic heart failure [ 9–13].

## Recommendation 2: Establish Formal Means of Setting Priorities for Conducting Large Clinical Trials

Uncovering confounders and being on guard against conventional “wisdom” can help “get future trials right,” but that still leaves open the question of whether to conduct a trial in the first place. The goal of a clinical trial is to benefit public health, usually by testing an intervention to reduce the morbidity or mortality of a disease. But it is clear that other priorities affect decision making—such as those listed in Box 2.

The strength of existing evidence is the most obvious and logical basis for the decision to launch a clinical trial. There is general agreement that the well-designed, large randomized double-blind and placebo-controlled trial is at the top of a hierarchical ladder of evidence in relation to the assessment of the effects of interventions. It is followed, in order of diminishing strength, by evidence from smaller randomized controlled trials, uncontrolled trials, observational studies, case studies, and, at the bottom, logical constructs (opinion) and anecdotes [ 14]. Whatever evidence exists at any level in the hierarchy needs to be weighed in the process of decision making.

However, this analysis of the literature may also involve other considerations, such as the level of risk compared with potential benefit, whether side effects are minor or major, the cost or feasibility of the intervention, and so on. The result is that ultimate decision and policy making may not always be completely logical, consistent, and transparent. These worrisome issues triggered considerable discussion at the meeting and inspired further recommendations.

## Recommendation 3: Employ Meta-Analyses and Systematic Reviews to Enhance Means of Weighing Evidence, but Beware of Potential Systematic Biases

The two formal tools most commonly used today to obtain an accurate overview of the literature are meta-analyses and systematic reviews. Meta-analyses are a means of obtaining greater statistical power and precision for data analysis by combining results from smaller clinical trials or studies into a summary statistic as long as the data meet the analysts' selection criteria and the studies allow for comparability. Systematic reviews have been pioneered by such organizations as the Cochrane Collaboration ( http://www.cochrane.org), the Agency for Healthcare Research and Quality ( http://www.ahrq.gov/), and its US Preventive Services Task Force ( http://www.ahrq.gov/clinic/uspstfix.htm), and it is suggested that systematic reviews be employed in the design and interpretation of all clinical trials [ 15, 16]. For each systematic review, the reviewers develop a set of criteria and grade the strength of each study selected for review. The individual grades in the aggregate determine the overall level of evidence: e.g., strong, weak, insufficient. Even using these more objective approaches, however, there is a danger that biases can influence the review. To deal with bias and inconsistencies across grading systems, an international GRADE Working Group ( http://www.GradeWorkingGroup.org) has developed a system that explicitly states the factors and methods used in making judgments [ 17].

And while one would hope that consistent results of multiple meta-analyses of smaller randomized studies would be confirmed in large clinical trials, data from a dozen large (more than 1,000 individuals) randomized clinical trials showed that 35% of the time, the outcomes were not predicted accurately by previously published meta-analyses [ 18]. Even outcomes of megatrials themselves on the same topic are not always consistent. In one study of pairs of large trials on the same topic, the results of 79 out of the 289 pairs (27%) differed in statistically significant ways [ 19].

## Recommendation 4: Give Equal Weight to Determining Safety and Efficacy in Clinical Trials

In September 2004, Merck (Whitehouse Station, New Jersey, United States of America) withdrew its cyclooxygenase-2 inhibitor rofecoxib (Vioxx) from the market because clinical trials showed increased risk of heart disease and stroke among long-term users of the pain drug [ 20, 21]. Concerns about rofecoxib and other cyclooxygenase-2 inhibitors led an expert committee convened by the Food and Drug Administration (FDA) to recommend that the Pfizer (New York, New York, United States of America) drugs celecoxib (Celebrex) and valdecoxib (Bextra) remain on the market, but no longer be advertised directly to consumers, and carry stringent “black box” warning labels about cardiovascular risks [ 22]. The FDA subsequently asked Pfizer to withdraw Bextra from the market [ 23]. In another instance of safety precautions, the FDA issued an advisory in October 2004 stating that selective serotonin reuptake inhibitor drugs used to treat depression may pose a suicide risk in adolescents [ 24].

Safety is a growing concern today, insofar as many clinical trials aim at preventing disease or forestalling disease progression and late-stage complications in people with chronic diseases. Because such trials often require large sample sizes and long follow-up to achieve definitive health outcomes, there is sometimes pressure to use surrogate (intermediate) end points to decrease trial size and duration. The results of a clinical trial of limited duration in relatively healthy adults can then translate to a recommendation for lifetime use of a drug (e.g., statins, antihypertensives) for an individual who may be older and less fit than the trial participants. Concerns about the risks of the lifetime use of drugs led many meeting participants to propose (1) longer trials, or at least longer-term follow-up, (2) empowering the FDA to conduct better postmarketing surveillance, and (3) broadly disseminating information on adverse events.

## Recommendation 5: Validate Biomarkers and Surrogate End Points before Basing Policy Guidelines for Public Health on Them

The shift in patterns of disease from acute infections to the chronic degenerative diseases affecting older populations has expanded the market for drugs. It has also created a growth industry in biomarkers, since the latter have the potential to allow for smaller trials of shortened duration. Broadly defined, a biomarker is “a characteristic that is objectively measured and evaluated as an indicator of normal biological processes, pathogenic processes, or pharmacological responses to a therapeutic intervention” (as defined at the 1999 NIH/FDA conference “Biomarkers and Surrogate End Points: Advancing Clinical Research and Applications” and stated in [ 25]).

## Screening Tests

A biomarker associated with a risk for disease is a candidate for a screening test. A case in point is the prostate-specific antigen (PSA). Tests showing high serum PSA values in men with prostate cancer drove clinical decisions for biopsy and subsequent medical or surgical treatment. But did the men benefit? The question remains open, but the harms are inescapable.

### The Prostate Cancer Prevention Trial.

This randomized placebo-controlled clinical trial enrolled nearly 19,000 men, 55 or over, with PSA levels of 3 ng/ml or under [ 26]. The men in the trial were treated with the 5-alpha reductase inhibitor finasteride, which lowers testosterone levels, or a placebo, and treatment was followed with periodic PSA tests and digital rectal exams for seven years. All were offered biopsy at the trial's end. The prevalence of prostate cancer in the finasteride group was 18.4%, compared with 24.4% in the placebo group ( *p* < 0.001). However, more than half of the men found with cancer in the placebo group had had a normal PSA and digital rectal examination throughout the trial. In a follow-up study of men in the placebo group, investigators concluded that there is no cutpoint of PSA such that higher scores are strongly associated with higher risk for clinically important prostate cancer and lower scores with lower risk; there are too many false positives and false negatives at every score [ 27].

The PSA test provides some important object lessons. It can detect a broad spectrum of prostate cancers. However, prostate cancers are a heterogeneous group—ranging from rapidly progressing and aggressive to slowly progressive or nonprogressive. Treating the last group may lead to the erroneous conclusion that these patients were “cured” by screening and treatment. If unscreened and untreated, these same men might simply have gone on to die from other causes—at the same point in time. Early diagnosis based on screening may also lead to the erroneous assumption that screening increases true survival time, since survival is measured from the time of diagnosis—longer in the case of the screening diagnosis compared with the time when the patient becomes symptomatic (the “lead time” bias).

## Surrogate (Intermediate) End Points

Biomarkers can also serve as “surrogate” or “intermediate” end points, instead of true health or clinical outcomes (how the patient feels or functions, or whether he or she survives). The virtue of a surrogate end point is that it provides a window at an intermediate point *t* in the trial, short of the true health or clinical outcome, and can serve as a bellwether that indicates whether treatment *x* is working or not, thus saving both time and money. But that depends on the validity of the surrogate. And validity is not easy to establish, as this example illustrates.

### Diabetes Control and Complications Trial.

Diabetic retinopathy with severe visual impairment or blindness is one of the long-term sequelae of diabetes. The Diabetes Control and Complications Trial was a randomized multicenter trial of 1,441 patients with diabetes to determine if intense monitoring of glucose and the use of an insulin pump would reduce the risk of retinopathy, inter alia [ 28]. The development of microaneurysms was chosen as a surrogate marker, since they are associated with vision loss. Early trends indicated an increase in microaneurysms and could have led to premature termination of the trial. But longer-term follow-up showed definite reduction in visual impairment, and the trial was appropriately ended at that point since it demonstrated a health benefit.

Similar cautionary tales can be told about the use of surrogates in other trials. A trial comparing continuous oxygen therapy with nocturnal oxygen only in patients with chronic obstructive pulmonary disease used a number of surrogate markers (e.g., hematocrit, cardiac index, pulmonary vascular resistance) to monitor effects and seemingly indicated no deleterious effects of the nighttime-only regimen. Nevertheless, the patients with continuous oxygen therapy had lower mortality than the group on the nocturnal regimen only [ 29].

Recognizing their potential value, several methods for the validation of biomarkers and surrogates were proposed at the meeting. One entails the use of hazard rates. The hazard rate is the risk of an event (such as death) at a given point in time in a clinical trial and can be computed for the experimental and control groups in the trial. The hazard rate for the experimental group divided by the hazard rate for the controls defines the “hazard ratio.” If this fraction is greater than one, the chances of succumbing to the health risk (such as death) increase with the treatment; if the ratio is less than one, the chances of the health risk decrease with treatment. The hazard rate framework could be used to establish, at the strongest, a causal link between a surrogate and the true clinical or health end point (essentially establishing that the surrogate captures or mediates the relationship between the treatment and the true end point), or less stringently, a strong association [ 30]. Also strengthening the case for validity would be corroborative findings from meta-analyses of smaller trials of a surrogate in relation to a given therapy. Other tests of validity for biomarkers invoke statistical measurements indicating that the marker demonstrates high sensitivity and specificity or has high predictive value, in an independent test sample [ 31].

To validate surrogate end points, a “validity” trial in which both surrogate and true end points are observed should be conducted, one in which it can be concluded that the inferences about the intervention were the same, whether based only on the surrogate or only on the true end point.

## Recommendation 6: Employ the Technologies of the “-Omics” Revolution and Systems Biology Approaches, but with Caution

The 21st century is seeing the growth of research on risk factors for disease and increased public interest in “wellness” and disease prevention. The tools for this research are the new technologies and databases of the “-omics” revolution: genomics, proteomics, metabolomics. The use of microarray technologies to determine patterns of genes activated in cells affected by disease compared with those in normal cells is a case in point—an exciting tool, yes, but one to be used with care, given the tendency to find patterns where none exist [ 32, 33]. Concerns about the credibility of positive findings in “-omics” searches have led some statisticians to propose a rethinking of such venerable measures of significance as *p* < 0.05 and to consider refinements in data analysis to guard against false positive results [ 34].

## Recommendation 7: Communicate Results of Clinical Trials in an Accurate and Timely Manner

The results of clinical trials are of little benefit to the public, patients, practitioners, or policy makers unless they are reported clearly and in a timely fashion. Some sponsors are reluctant to publish negative or adverse findings [ 35]—as are some peer-reviewed journals, leading to a skewing of the literature—although there are signs that this may be changing. Recently, a number of major medical journals have agreed to publish articles on clinical trials only if they have been previously registered in an accessible database. (The Uniform Requirements established by the International Committee of Medical Journal Editors stipulate that as of September 2005 participating journals will consider for publication only those clinical trials that have been properly registered with a publicly available registry. See the Web site: http://www.icmje.org.)

In any case, what is said sometimes reveals a spin on data to favor a particular point of view. Results of a new therapy may be stated in terms of a relative percent improvement—but a percent of what? The base rate (the number of clinical events or the event rate) is often not stated. Reporting a relative percent improvement often exaggerates apparent benefits compared with reporting absolute rates with (versus without) the intervention.

Examples of such misleading “data framing” presented at the meeting included an ad for an anti-osteoporosis drug that claimed that individuals taking the drug in a year's trial experienced a 68% reduction in clinical vertebral fractures over a comparable group on placebo. However, a look at the actual figures (see sidebar) showed that there were five fewer fractures per 1,000 women among the drug users—leaving a far different impression of the magnitude of benefit. Framing can be particularly misleading when benefits are reported as relative risks and harms are reported as absolute risks.

Peer-reviewed articles may also be misleading. A paper in a leading medical journal reporting on a population-based case-control study of breast cancer stated that women who used aspirin over a five-year period had a 20% reduction in breast cancer [ 36]. Here the 20% represents the relative risk, derived by dividing the 1.6% risk of breast cancer reported for women who took aspirin by the 2.0% risk of breast cancer in women not taking aspirin and subtracting the fraction from one: 1 - (1.6%/2.0%) = 1 - 0.8 = 0.2 = 20%. (Since then, a large randomized placebo-controlled trial has shown no benefit of aspirin on breast cancer incidence [ 37].)

The “take-home message” from these examples is to search for the meaning of the data behind the headlines—and for investigators to report more useful statistics, such as basic information on event rates and the use of absolute rates [ 38]. In this regard, the work of the CONSORT (Consolidated Standards of Reporting Trials) group in developing a checklist and flow diagram to improve the quality of reports on randomized clinical trials is highly commendable (see http://www.consort-statement.org).

## Recommendation 8: Establish Two-Way Communications with Communities, Consumers, and Patient Advocacy Groups in Developing, Implementing, and Reporting Clinical Trials

That message is taken very seriously by two other groups of key players in communicating the results of clinical trials: reporters and patient advocacy groups. The best reporters do much more than act as passive conveyors of news releases about scientific findings; they serve as “honest brokers” of the evidence and provide context for new findings.

Patient advocacy groups play a different role from journalists, since they have an interest in promoting research and communicating results. Noteworthy among such groups has been the AIDS activist organization, Act Up. A representative of the group offered a cautionary tale about surrogate end points. To speed up the development of AIDS drugs in the early 1990s, some advocates urged use of an increase in CD4 T cell counts as a surrogate for efficacy in clinical trials—which, in the case of dideoxyinosine, was a mistake. Changes in the CD4 counts did not always accurately predict health outcomes and survival.

## Recommendation 9: Establish Criteria to Inform Public Policy and Decision Making

Given the potential lifesaving or life-enhancing benefits of a clinical trial, formal steps are needed to move outcomes and information into public health policy and decision making. Transparency and consistency in the process are key elements. For government-sponsored trials, the agency sponsoring the trial bears the responsibility for the timely (1) publication of findings, (2) communication to the public and health professionals, and (3) provision of information to the Secretary, Health and Human Services, and the heads of other public health agencies as appropriate. Of course, responsibility for application to public health policy rests with elected representatives of the public.

## Conclusion: Prospects for the Future

In spite of the shortcomings in observational studies that have led to mistakes in the interpretation or application of evidence, there was an overall air of optimism at the meeting that taming the new technologies and applying strategies for rigorous statistical and study design may help resolve some of the uncertainties that bedevil clinical trials today. Greater knowledge of pharmacogenetics and metabolomics, and a better understanding of how genes interact with each other and with environmental variables, may lead to new designs for clinical trials. Medicine may see an acceleration of the movement toward prevention of disease and maintenance of health and well-being. All this will be welcome news to the public, advocacy groups, the media, policy makers—and scientists themselves, who are among the first to admit, as Aristotle said, that “the investigation of truth is in one way hard, in another, easy.”

**Box 1. Recommendations Arising from the Meeting**

- Be aware of and eliminate bias and confounders to the extent possible.
- Establish formal means of setting priorities for conducting large clinical trials.
- Employ meta-analyses and systematic reviews to enhance means of weighing evidence, but beware of potential systematic biases.
- Give equal weight to determining safety and efficacy in clinical trials.
- Validate biomarkers and surrogate end points before basing policy guidelines for public health on them.
- Employ the technologies of the “-omics” revolution and systems biology approaches, but with caution.
- Communicate results of clinical trials in an accurate and timely manner.
- Establish two-way communications with communities, consumers, and patient advocacy groups during development, implementation, and reporting of clinical trials.
- Establish criteria to inform public policy and decision making.

**Box 2. Factors Affecting the Decision to Conduct a Clinical Trial**

- Strength of existing evidence: promising, but not conclusive data from pre-clinical and observational studies/small trials
- Potential impact on public health and/or medical science
- Therapeutic equipoise: difficulty deciding which of two therapies is better without a head-to-head test
- Portfolio balance: a research organization may adjust its priorities to give more (or less) attention to specific areas to achieve optimal use of its resources
- A potential for runaway practice, e.g., a new diagnostic test that is being widely marketed, but has not been adequately evaluated
- Social/political context/pressures—from legislators, community leaders, patient advocacy groups

**Presenting Relative Risks Alone Can Be Misleading**

“… a 68% reduction!”

This ad for an antiosteoporosis drug claimed that individuals taking the drug in a year's trial experienced a 68% reduction in clinical vertebral fractures over a comparable group on placebo.

What were the actual figures? The rate of fractures among placebo users was 0.738%. The rate of fractures among the drug users was 0.238%. Thus, the absolute risk reduction was 0.738% - 0.238% = 0.5%, which translates to five fewer fractures per 1,000 women—hardly headline news. But by presenting the data in terms of relative risk reduction—relative risk reduction = 1 - 0.238%/0.738% = 0.678 (approximately 68%)—the impressive 68% figure was advertised. Computations of relative risk reduction will always appear impressively large when actual event rates are low.

## Abbreviations

FDA: Food and Drug Administration
HRT: hormone-replacement therapy
NIH: National Institutes of Health
PSA: prostate-specific antigen
